# Factors Related to the Quality of Life in Family Carers of People With Dementia: A Meta-Analysis

**DOI:** 10.1177/0891988720924713

**Published:** 2020-05-12

**Authors:** Milena L. Contreras, Eneida Mioshi, Naoko Kishita

**Affiliations:** 1 School of Health Sciences, 6106 University of East Anglia, Norwich, United Kingdom

**Keywords:** dementia, family caregivers, quality of life, QoL, Alzheimer’s

## Abstract

**Objectives:**

This meta-analysis aimed to (1) quantitatively synthesize evidence of factors related to the quality of life (QoL) of family carers of people with dementia and (2) explore moderating factors that may influence the strength of the relationship between such potential predictive factors and carer QoL.

**Methods:**

Studies that investigated correlations between patient/carer factors and QoL in unpaid family carers of people with dementia and were published in English, Spanish, Portuguese, or Japanese were included.

**Results:**

Thirty-three studies were identified. The pooled correlations with carer QoL (effect size) were significantly large for depression (−0.58), significantly moderate for subjective burden (−0.47), and significantly small for people with dementia’s neuropsychiatric symptoms (−0.24). These results indicated to be robust in the context of publication bias. The results of subgroup analyses demonstrated the social and economic development status of the country where study participants resided did not moderate these effects.

**Conclusion:**

Carer depression, subjective burden, and people with dementia’s neuropsychiatric symptoms may play a critical role in maintaining QoL of family carers regardless of the social and economic circumstances.

## Introduction

The number of people living with dementia worldwide is currently estimated at 35.6 million, and this number is expected to double by 2030 and more than triple by 2050.^1^ Dementia is one of the most expensive health conditions, and the current annual worldwide cost of dementia is estimated to be US$818 billion.^2^ As such, dementia is considered as one of the greatest health challenges we face today.

Dementia is a progressive condition, and while some individuals maintain their independence for many years, many require progressively more support with daily activities, particularly in the later stage of the condition.^3^ Family members are considered as a primary resource for this type of care in many countries. For example, in the United Kingdom, people affected by dementia and their relatives are currently shouldering two-thirds of all dementia care costs, saving the UK economy billions each year.^4^ In Latin-American countries, such as Brazil, there are fewer health care services specialized in dementia, which reinforces the belief that families should be responsible for the person with dementia.^5^ The lack of provision of dementia services within the public health care system is also common in Asian countries such as China, and as a consequence, families take over the significant caring role.^6^

These suggest that unpaid family carers are an essential taskforce in caring for people with dementia worldwide. Therefore, this review focused on unpaid family carers (ie, informal carers) who are characteristically different from formal carers (ie, health care professionals) paid to provide essential care.

Caring for someone with dementia can be physically and emotionally demanding, and it can seriously affect the social, psychological, and physical well-being of the family carer.^7,8^ The previous literature demonstrates that poor carer quality of life (QoL) is likely to be associated with poorer QoL for the person with dementia^9^ and with higher economic costs.^10^

Quality of life is a term frequently used in the literature, but, to date, there is no consensus about how to best define and assess QoL in family carers of people with dementia.^11,12^ The World Health Organization (WHO) defines it as the individual’s perception of their position in life in relation to their goals, expectations, standards, and concerns, according to the culture and value systems in which they live. General QoL includes several aspects such as psychological state, physical health, level of independence, personal beliefs and spirituality, social relationships, and environment.^13^ There is another important concept of QoL often used in the literature that is the health-related QoL (HRQoL). Health-related QoL refers to the components of QoL that are directly and indirectly affected by health, disease, disorder, and injury, and therefore, HRQoL often overlaps with the concept of health status.^14,15^

In the past 10 years, there have been emerging studies, which have developed more specific instruments to measure carer QoL.^11,16,17^ Early carer studies predominately used general QoL and HRQoL measures. The use of general QoL and HRQoL instruments with older carers can be problematic, as some aspects of these types of QoL (eg, level of independence) could be affected by their age-related factors such as changes in physical conditions.^18^ In this regard, these types of instruments have been criticized for lacking validity and not being sensitive enough to measure the psychological consequences and positive aspects of caring.^11,19^ In this meta-analysis, we defined the QoL of carers in a broader sense and included all types of QoL measures to provide a wider understanding of the potential impacts of different factors on carer QoL.

The national guidelines and policies such as the UK Government’s action plan^20^ emphasize the need for focusing on early interventions for carers to support them maintaining their QoL. For this reason, it is fundamental to identify the modifiable factors that may affect the family carers’ QoL in order to guide the formulation and delivery of policy, treatment, care, and support to improve this crucial outcome.^21^

Previously, there have been 3 review studies that have examined factors associated with the QoL of family carers of people with dementia. The first systematic review conducted by de Oliveira et al, which solely focused on examining the association of carers’ advanced age with their QoL, demonstrated that carer’s advanced age was associated with low levels of their QoL.^22^

The second study, an integrative review conducted by Pereira and Soares and published in Portuguese, found that both factors related to carers themselves (eg, having depression, poor sleep quality, preexisting health problems, social support received, leisure activities, having received interventions, or training for carers) and people with dementia (eg, dementia type and neuropsychiatric symptoms) can influence the QoL of family carers.^23^

The most recent systematic review conducted by Farina et al found that having better physical and mental health was the factor most strongly associated with having a better QoL. They also found that greater carer independence (eg, activities and time not spent on caring duties) was positively associated with better QoL and that carers who lived with the care recipient had poorer QoL than those who did not. The health status of the people with dementia and their behavioral and psychological symptoms also seem to be detrimental to carer QoL.^21^

These 3 reviews highlighted that both carer- and patient characteristics could be potential predictors of carer QoL. However, these reviews have some methodological limitations. First, all reviews only included studies written in English, which might have induced a bias in the findings. One of the reviews^22^ only included studies that targeted carers aged 60 years or older, and all included studies were carried out in developed countries, and thus, the generalizability of the findings may be limited due to selection bias. When comparing the distribution of the total costs of dementia worldwide, 87% is currently spent in high-income countries, despite the fact that the contribution of informal carers is expected to be greatest in developing countries.^2^ It is, therefore, important to explore the impact of dementia across countries with different economic development status. Another limitation is that the second review by Pereira and Soares did not employ a systematic approach, but it was rather an integrative review using purposive sampling. Therefore, the findings could be prone to researcher bias.^23^

Large heterogeneity in the study designs was also evident across all 3 reviews. The authors combined correlational and regression studies^21-23^ and included interventional and cross-sectional studies^23^ or quantitative and qualitative studies^21^ in their single purposive sampling review. As a result, the included studies were completely heterogeneous, making it difficult to draw a robust conclusion.

Moreover, although the most recent review by Farina et al was published in 2017, the literature search was conducted in November 2015. Taking into consideration that in recent years, there has been an increasing interest in dementia care research,^24^ it is expected to find a larger number of articles over the last few years. As such, an updated review could address previous limitations and enhance our understanding of factors associated with carer QoL.

To overcome the aforementioned limitations and clarify the current state of the evidence base, an updated review using a meta-analytic approach was conducted with the following objectives:

1. To quantify the point estimate of effect size between carer QoL and different types of independent variables including those related to carers themselves (eg, carer depression) and people with dementia (eg, neuropsychiatric symptoms); and
2. to explore factors that may moderate the strength of such relationship, including the development status of the country and types of tools used to assess the constructs of interest.

## Methods

This meta-analysis adhered to the Preferred Reporting Items for Systematic Reviews and Meta-Analyses (PRISMA) guidelines.^25^ The PRISMA checklist is included as a supplementary file (see Supplementary Table 1).

### Eligibility Criteria

The review included quantitative articles published in peer-reviewed journals or academic reports (eg, PhD thesis). Only cross-sectional and longitudinal studies were eligible for the review.

In order to be eligible for the current review, the study had to (1) recruit unpaid family carers of people with dementia; (2) use a validated measure of generic, health-related, or care-related QoL to assess QoL in family carers as a dependent variable; (3) be published in English, Spanish, Portuguese, or Japanese; and (4) report a Pearson or Spearman correlation between the dependent variable (ie, carer QoL) and independent variables. Any types of independent variables were eligible for the review, including variables related to carers themselves (eg, carer depression) and people with dementia (eg, neuropsychiatric symptoms).

### Information Sources

The databases of PubMed, PsycINFO, and Scopus were searched to identify relevant published articles. ProQuest was used to search unpublished doctoral thesis, and Lilacs and Scielo were used to search for studies from Spain and Latin America.

### Search

The search was conducted by the first author (M.C.) using the key words and search strategies outlined in Supplementary Table 2. Manual searches in the reference lists of relevant systematic reviews and articles were also completed to identify any potential missing articles. No date restriction was applied to the search for studies.

### Study Selection

Search results were merged using EndNote software, and duplicate articles were removed. All the titles and abstracts were screened for eligibility by the first author (M.C.), whereby clearly irrelevant articles were excluded. Following the initial screening, full-text articles were reviewed by 2 authors (M.C. and N.K.) independently using a structured checklist. The Kappa coefficient for the interrater agreement was .84, indicating almost perfect agreement.^26^ Disagreements between 2 coders were resolved through discussions.

### Data Collection Process

The first author (M.C.) developed an electronic data extraction sheet that was pilot tested on a randomly selected study by 2 authors (M.C. and N.K.). Following this, the electronic form was refined accordingly. To minimize bias, data extraction was conducted on the first 5 selected studies by 2 authors (M.C. and N.K.) independently. No discrepancies were identified during this pilot phase. Following this, the first author (M.C.) and a research assistant independently extracted data from the remaining studies. The agreement rate between the 2 coders was 90.3%, indicating almost perfect agreement.

### Data Items

For each included study, information was recorded on (1) study characteristics (the country where the study was conducted and study design); (2) sample characteristics (number of participants, age, gender, relationship with the person with dementia, and the average length being a carer); (3) dementia type of the carer recipient; (4) measures used to assess carer QoL; (5) measures used to assess independent variables; and (6) correlation coefficient between carer QoL and the independent variables. If relevant information was not provided in the selected studies, it was considered as “not reported,” and the authors did not contact researchers for further clarification.

### Risk of Bias in Individual Studies

The Appraisal of Cross-sectional Studies^27^ was used to assess the risk of bias in each included study. This tool consists of 20 items, which assess different aspects of the methodological quality and reporting quality such as appropriateness of study design and target population, measurement validity and reliability, appropriateness of interpretation of results, and justification of conclusion. The Appraisal of Cross-sectional Studies does not include a numerical scale that can be used to produce a quality assessment score; instead, it aims to measure the individual characteristics of a study cumulatively.^28^ The first author and a research assistant assessed the risk of bias independently, and disagreements were discussed. The Kappa coefficient for the interrater agreement was 0.56 indicating moderate agreement between the raters.^26^

### Summary of Measures and Synthesis of Results

The entire analysis was conducted using Comprehensive Meta-Analysis software version 3.^29^ There are no simple criteria in terms of how many studies are needed to calculate the meaningful pooled effect size. However, the combination of very few studies with very different characteristics makes any kind of synthesis untenable in most cases.^30^ In this study, the meta-analysis was conducted only when the correlation coefficient between carer QoL and the targeted independent variable was available from more than 3 studies (ie, if only 2 studies reported the correlation coefficient between carer QoL and the targeted independent variable and then quantitative synthesis was not performed).

The correlation coefficient from included studies was transformed to corresponding Fisher scores to estimate a pooled effect size and its 95% confidence intervals (CI) for each independent variable. A fixed-effect model was used to provide a pooled estimated effect for each independent variable, and a test for heterogeneity was performed using the *Q*-statistic and the *I*^2^ statistic. Where there was evidence of heterogeneity, a random effects model was used. Estimated effect sizes of <0.09 were considered negligible, 0.10 to 0.29 small, 0.30 to 0.49 moderate, and >0.50 large.^31^

If the correlation coefficient for the same independent variable was reported from 2 or more independent samples within a single study, they were treated as separate studies for the purpose of analyses. For example, the correlation coefficient for the same independent variable was reported separately for female and male samples in one study^32^ and for carers of people with mild, moderate, and severe dementia in another study.^33^ When the correlation coefficient for the same independent variable was reported for each subscale of the QoL measure rather the total QoL score within a single study,^34^ correlation coefficients were combined by calculating the mean of effect sizes across subscales to produce a single effect size.^35^ The “total QoL score” was used when possible.^36^

### Risk of Bias Across Studies

To assess publication bias, the trim and fill method^37^ was used to estimate how many studies could be missing from each meta-analysis and calculate adjusted effect-size estimates. Rosenthal’s Fail-Safe N^38^ was used to calculate the number of missing studies needed to be included in the analysis to reduce the overall effect size to a nonsignificant level. If only a few studies are required to nullify the observed effect, the observed overall effect may not be robust.^35^

### Additional Analyses

For those independent variables, which demonstrated a significant heterogeneity, a series of subgroup analyses were planned to examine the possible sources of variance. Initially, a series of subgroup analysis using the following moderators were planned: (1) the development status of the country as defined by the Human Development Index (HDI) category (low, medium, high, and very high), which is a summary measure of a country’s overall achievement in its social and economic dimensions (ie, health, education, and standard living)^39^; (2) types of measures used to assess carer QoL; (3) types of measures used to assess the independent variable; (4) the relationship with the person with dementia; (5) dementia type of the care recipient; (6) carer’s gender; and (7) average length being a carer. However, the latter 4 moderators (ie, relationship, dementia type, gender, and length as a carer) were not reported consistently in many of the included studies or seemed to be similar across the included studies that did report. Therefore, it was not possible to conduct the subgroup analyses using these 4 moderators.

## Results

### Study Selection

**Figure 1.**
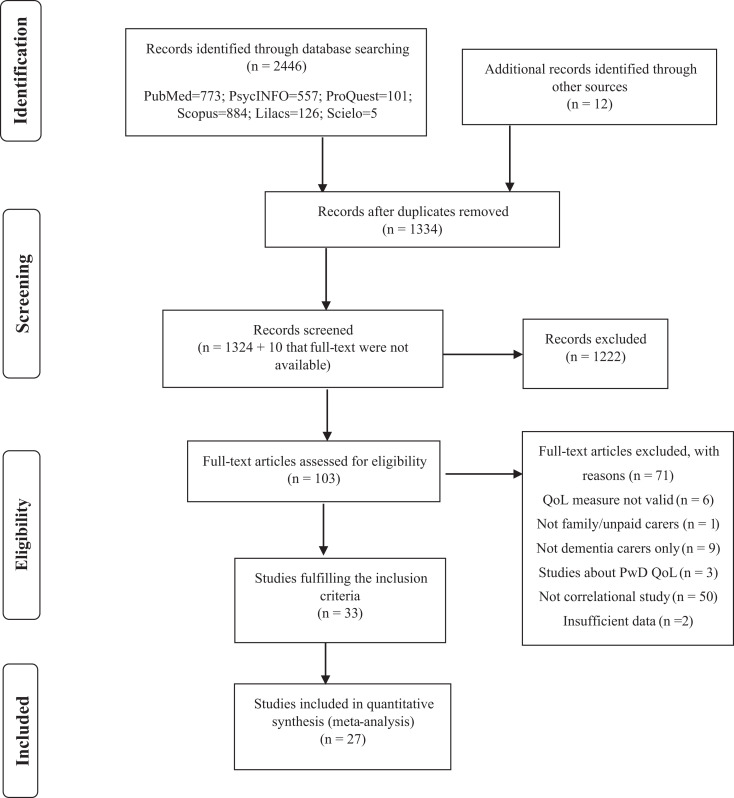
Preferred Reporting Items for Systematic Reviews and Meta-Analyses (PRISMA) flowchart of the selection of studies.

The search was conducted on May 30, 2018, and a total of 2458 articles were found. After deleting 1124 duplicated articles, 1334 titles and abstracts were examined by the first author (M.C.). One hundred and two studies were identified as relevant for the meta-analysis, and the full text were reviewed by the 2 coders (M.C. and N.K.) independently. From the 102 full texts reviewed, 33 fulfilled the inclusion criteria, and data were extracted from each study. However, only 27 were included in the final meta-analysis (see Figure 1). The remaining 5 studies did report correlations between QoL and some independent variables, but data for the same independent variable were not available from more than 3 studies. Thus, these 5 studies were not included in the quantitative synthesis.

### Study Characteristics

#### Participants

The characteristics of included studies are presented in Table 1. The total number of carers was 6177. The majority of studies recruited carers from Europe (study n = 12), North America (n = 8), and South America (n = 8). There were fewer studies which recruited carers from Asia (n = 4) and Oceania (n = 1). More than 65% of carers were females in the majority (over 70%) of the studies included (n = 24). Over 75% of the studies (n = 26) recruited people over 55 years old, and 78% of studies only recruited carers with Alzheimer’s disease (n = 26). This diagnosis was the most prevalent in the remaining studies. Eight studies did not report the type of dementia of the care recipient. These results suggest that carers recruited in the identified studies were predominantly females over 55 years old looking after a family member with Alzheimer’s disease.

**Table 1.** Characteristics of Included Studies.^a^

Authors	Country	Sample	Relationship with patient, %	Average length being carer, years	Care recipient characteristics (diagnosis, severity %)	Carer QoL measures	Variables correlated with QoL
1. Andreakou (2016)	Greece	155 carers	Spouse: 38.00	4.6	Alzheimer: 100.0	SF-36 (mental and physical components)	**Depression (ZDRS)**
		Female %: NR	Daughter/son: 48.40		Mild: 22.6		
		Mean age (SD): 58.1 (13.4)	Siblings: 2.60		Moderate: 54.8		
			Other: 11.60		Severe: 22.6		
2. Araujo de Amorim (2017)	Brazil	41 carers	Spouse: 34.10	4.8	Alzheimer: 100.0	WHO-QOL-BREF	**Social Skills**
		Female %: 87.8	Daughter/son: 56.09		Severity: NR		
		Mean age (SD): 61.09 (13.4)	Other: 9.81				
3. Borghi (2011)	Brazil	50 carers	Spouse: 16.00	4.63	Alzheimer: 100.0	QoL-AD	**Carer-rated PwD QoL (QoL-AD)**
		Female %: 82.0	Daughter: 60.0		Severity: NR		
		Mean age (SD): 53.83 (14.52)	Other: 24.00				
4. Coen (1999)	Ireland	50 carers	Spouse: 46.00	2 (Median)	Alzheimer: 100.0	Evaluation of Individual Quality of Life—Direct Weighting (SEIQoL-DW)	**Perceived Burden (ZBI); Well-being;** Social support; Behavior disturbance (DBD); Cognitive functioning; Functional status; Carer-rated patient QoL (QoL-AD)
		Female %: 72.0	Daughter/son: 44.00		Mild: 66.0		
		Mean age: 56.5	Sibilings: 2.00		Moderate: 22.0		
			Other: 8.00		Severe: 12.0		
5. Conde-Sala (2010)	Spain	251 carers	Spouse: 44.60	NR	Alzheimer: 100.0	SF-12 (mental component)	**Daughter-rated patient QoL (QoL-AD)**; Wives-rated patient QoL (QoL-AD); Husbands-rated patient QoL (QoL-AD); Son-rated patient QoL (QoL-AD)
		Female %: 66.1	Daughter/son: 55.30		Mild: 10.36		
		Mean age (SD): Spouse: 73.6 (7.4); Child: 49.3 (7.2)			Moderate: 68.92		
					Severe: 20.72		
6. Creese (2008)	Canada	60 carers	Spouse: 100	4.61	Alzheimer: 100.0	SF-36 (mental and physical components)	Current sleep quality; Change in sleep quality; Frequency of nocturnal disruptions; Current sleep quality; Change in sleep quality; **Frequency of nocturnal disruptions**
		Female %: 68			Severity: NR		
		Mean age (SD): 73.65 (9.26)					
7. Crellin (2015)	United Kingdom	289 carers	Spouse: 63.3	4.4	Alzheimer: 51.0	SF-12 (mental and physical components)	**Positive impact; QoL physical component score (SF-12); Self-efficacy for obtaining respite; Self-efficacy for responding to disruptive behaviors; Self-efficacy for controlling upsetting thoughts; Self-efficacy for managing neuropsychiatric symptoms; Quality of support,** Emotion-focused coping; **Problem focused coping; Dysfunctional coping; PwD neuropsychiatric symptoms (NPI);** PwD Cognitive funtioning; **PwD ADL**
		Female %: 68.2	Adult child/other family: 34.9		Vascular: 18.6		
		Mean age (SD): 66.7 (12.3)	Other: 1.7		Others: 30.4		
					Mild: 63.0		
					Moderate: 27.0		
					Severe: 10.0		
8. Feast (2017)	United Kingdom	157 carers	Spouse: 53.55	NR	Diagnosis: NR	EQ-5D	BPSD-related distress; Frequency of BPSD; **Relationship quality**; Carer competence; **Carer guilt;** Carer-rated patient QoL (EQ-5D); **Burden (The relative stress scale)**; Reactivity to BPSD
		Female %: 70.96			Severity: NR		
		Mean age: 66.34					
9. Häusler (2016)	Germany	82 carers	Spouse: 100	NR	Alzheimer: 78.05	WHOQOL-BREF	**Perceived Stress**
		Female %: 60.97			Vascular: 18.6		
		Mean age (SD): 73.02 (6.68)			Lewy bodies:		
					Others: 30.4		
					Severity: NR		
10. Jackson (2009)	United Kingdom	132 carers	Spouse: 36.00	NR	Alzheimer: 100.0	WHO-QOL-BREF	Activities of Daily Living; **Memory and Behaviour Problems (MBPC-1990 R)**
		Female %: 72.0	Offspring (or son		Severity: NR	Physical	
		Mean age (SD): 62 (13.4)	or daughter in law): 44.00			Psychological	
			Siblings: 4.00			Social	
			Other: 16.00			Environmental	
11. Kaufman (2010)	United States	141 carers	Spouse: 9.9	NR	Diagnosis: NR	Quality of Life Inventory (QOLI)	**Interpersonal Support tangible component; Interpersonal Support appraisal component; Interpersonal Support belonging component; Interpersonal Support self-esteem component**
		Female %: 85.1	Daughter/son: 58.9		Severity: NR		
		Mean age: 52	Other: 31.2				
12. Kim (2016)	South Korea	476 carers	Spouse: 67.7	4.3 ± 4.6	Diagnosis: NR	SF-36 (mental and physical components)	**QoL Mental component & Physical component (SF-36); Depression (BDI); Burden (ZBI) Extraversion;** Agreeableness; Conscientiousness; **Neuroticism**, Openness
		Female %: 67.7	Daughter/son: 37.9		Severity: NR		
		Mean age (SD): 57.4 (13.1)	Other: 42.5				
13. Kramer (1993)	United States	72 carers	Spouse: 100	4.75	Alzheimer: 100.0	The Quality of Life Index	PwD functional status ADL; PwD functional status instrumental ADL; **PwD Memory and behavior problems (MBPC)**; Caregiver age; Duration of caregiving; **Quality of prior relationship**; **Physical health**; Family income; **Social involvement satisfaction**; Appraisal of the stressfulness of ADL; **Appraisal of the stressfulness of IADL**; **Appraisal of the stressfulness of MBP**
		Female %: 100.0			Severity: NR		
		Mean age: 70.0					
14. Markowitz (2003)	United States	2477 carers	Spouse: 67.7	NR	Alzheimer: 100.0	SF-12 (mental and physical components)	**PwD disruptive behaviour (MBPC-R); PwD feelings of depression (MBPC-R); PwD Memory (MBPC-R);** PwD instrumental funtioning; PwD personal funtioning; **No hours per week providing care**; Caregiver’s age
		Female %: 77.7	Daughter/son: 37.9		Severity: NR		
		Mean age (SD): 58.8 (10.1)	Other: 42.5				
15. McConaghy (2005)	Australia	42 carers	Spouse: 54.76	5.45	Diagnosis: NR	SF-12 v2 Physical component	Coping; Burden (ZBI); Satisfaction with life
		Female %: 76.2	Daughter/son: 34.8		Mild: 40.9		
		Mean age (SD): 62 (13.2)	Other: 9.5		Moderate: 18.18		
					Severe: 40.9		
16. McLennon (2011)	United States	84 carers	Spouse: 100	4.6	Diagnosis: NR	SF-36 v2 (mental and physical components)	**Income; Duration of caregiving; Burden (ZBI); Finding meaning;** Education;
		Female %: 59.5			Severity: NR		
		Mean age (SD): 73.3 (10.5)					
17. Moreno (2015)	Colombia	102 carers	NR	3.9	Diagnosis: NR	SF-36 Physical functioning, Role-Physical, Vitality, Social functioning, Bodily pain and General Health components	**Satisfaction with life; Depression (PHQ-9); Burden (ZBI)**
		Female %: 81.4			Severity: NR		
		Mean age (SD): 58.4 (13.3)					
18. Nogueira (2015)	Brazil	54 carers	Spouse: 100	NR	Alzheimer: 100.0	QoL-AD	**PwD QoL (QoL-AD); Burden (ZBI); PwD functional status; PwD awareness of disease**
		Female %: 66.7			Moderate: 62.96		
		Mean age (SD): Males: 72 (13.6); Females: 67.6 (8.2)			Severe: 37.04		
19. Novelli (2010)	Brazil	60 carers	Spouse: 41.67	NR	Alzheimer: 100.0	QoL-AD (mild dementia and moderate dementia)	PwD cognitive function; PwD depression/mood; **PwD Insturmental ADL; PwD ADL; PwD behavioral disturbances (NPI); Carer depression/mood (GDS); PwD QoL self-reported; Carer-rated PwD QoL (QoL-AD)**
		Female %: 73.3	Daughter/son: 41.67		Mild: 50.0		
		Mean age (SD):	Sibilings: 13.33		Moderate: 50.0		
		Mild dementia: 59.5 (15.4)	Other: 3.3				
		Moderate: 60.1 (14.5)					
20. Papastavrou (2014)	Cyprus	76 carers	Spouse: 53.0		Diagnosis: NR	QoL-AD	**Burden (ZBI); Depression (CES-D);** ADL
		Female %: 75.0	Other: 47.0	1-2: 33.3	Severity: NR		
		Age%: <50: 18.0; 51-60: 25.0; 61-70: 29.0; >71: 21.0		3-4: 28			
				>5: 38.7			
21. Perrin (2014)	Colombia	90 carers	Spouse: 17.8	3.7	Alzheimer: 91.11	SF-36 (Values not available to conduct meta-analysis)	**Satisfaction with life;** Depression (PHQ-9)**; Burden (ZBI)**
		Female %: 64.4	Daughter/son: 22.2		Vascular: 4.44		
		Mean age (SD): 54.1 (11.5)	Sibilings: 60.0		Mixed: 2.22		
					Others: 2.22		
22. Santos (2014)	Brazil	88 carers	Spouse: 31.8	4.4	Alzheimer: 100.0	QoL-AD	Carer’s gender; Carer’s age; Carer’s schooling; **Burden (ZBI); Mood (BDI); Anxiety;** PwD gender; PwD age; PwD schooling; PwD marital status; **PwD age of onset; PwD duration of disease; PwD self-rated QoL (QoL-AD); PwD carer-rated (QoL-AD); PwD cognition; PwD depression; PwD funtional activities; PwD Neuropsychiatric symptoms (NPI)**
		Female %: 76.1	Daughter/son: 48.9		Mild: 48.9		
		Mean age (SD): 59.22 (13.8)	Other: 19.3		Moderate: 51.1		
23. Schiffczyk (2013)	Germany	194 carers	NR	NR	Alzheimer	QoL-AD	PwD cognitive symptoms; **Noncognitive symptoms of the PwD (Behave-AD)**
		Female %: 72.2			(most of them)		
		Mean age (SD): 69 (7.7)			Severity: NR		
24. Scholzel-Dorenbos (2009)	the Netherlands	87 carers	NR	NR	Alzheimer: 100.0	SEIQoL	**PwD cognitive symptoms; Burden (ZBI)**
		Female %: 47.0			Severity: NR		
		Mean age (SD): 72.2 (7.3)					
25. Shin (2005)	United States	62 carers	Spouse: 51.6	NR	Diagnosis: Alzheimer	QoL-AD	**PwD Neuropsychiatric symptoms (NPI); Caregiver distress**
		Female %: NR	Daughter/son: 33.9		Severity: NR		
		Mean age (SD): NR	Other: 14.5				
26. Takahashi (2005)	Japan	23 carers	Spouse: 78.3	3	Alzheimer: 73.9	WHO-QOL26	**Depression**
		Female %: 78.27	Daughter/son: 60.9		Vascular: 4.3		
		Mean age (SD): 61.1 (13.0)	Other: 17.4		Lewy bodies: 8.7		
					Frontotemporal: 8.7		
					Others: 4.4		
					Mild: 30.4		
					Moderate: 30.4		
					Severe: 3.1		
27. Takai (2011)	Japan	118 carers	Spouse: 55.1	NR	Alzheimer: 77.9	WHO-QOL26	PwD Cognitive function; PwD Cognitive and functional performance; **PwD Neuropsychiatric symptoms (NPI); Burnout; Depression (BDI-II)**
		Female %: 59.3	Daughter/son: 37.3		Vascular: 11.0		
		Mean age (SD): 60.9 (14.0)	Other: 7.6		Lewy bodies: 2.5		
					Frontotemporal: 4.2		
					Mixed: 4.2		
					Severity: NR		
28. Tay (2016)	Singapore	84 carers	Spouse: 7.1	NR	Alzheimer: 36.9	WHO-QoL-BREF	**Family burden (FBIS); Coping strategies Total; General perceived** self-efficacy**; Caregiver’s age; Patient’s age; Income**
		Female %: 69.0	Daughter/son: 83.3		Vascular: 27.4		
		Mean age (SD): 50.89 (10.6)	Other: 9.6		Mixed: 35.7		
					Mild: 59.5		
					Moderate: 40.5		
29. Thompson (2004)	United States	61 carers	Spouse: 100	5.3	Alzheimer: 100.0	SF-36 (Mental component)	**Natural killer cell number; Male Sense of coherence; Male Depression (CES-D); Male Stress; Female Sense of coherence; Female Depression (CES-D); Female Stress**
		Female %: 73.80			Severity: NR		
		Mean age:					
		Female: 69.7					
		Male: 71.4					
30. Valimaki (2009)	Finland	170 carers	Spouse: 100	NR	Alzheimer: 100.0	15D + 15D VAS	**PwD Cognitive function; PwD Neuropsychiatric symptoms (NPI);** PwD Cognitive function; Caregiver’s age; PwD age; **HRQoL VAS; Sense of Coherence; Distress; Depression (BDI);** Income**; Total amount of medication;** Years of education
		Female %: 62.9			Severity: Only Mild		
		Mean age (SD): 71.6 (7.2)					
31. Vargas Escobar (2010)	Colombia	192 carers	Daughter/son: most of them	NR, between 10-36 months	Alzheimer: 100.0	QoL (Betty Ferrell)	PwD functional dependency
		Gender: most of them women			Mild: 25.5		
		Age: 36-59 years old			Moderate: 45.8		
					Severe: 28.6		
32. Weisman de Mamani (2017)	United States	106 carers	Spouse: 14.2	NR	Alzheimer: 100.0	Quality of Life Inventory (QoLI)	**Expressed Emotion (EE) total; EE Emotional Overinvolvement; EE Criticism**
		Female %: 81.1	Daughter/son: 51.9		Severity: NR		
		Mean age (SD): 50.73 (12.7)	Sibilings: 1.9				
			Other: 32.1				
33. Zawadzki (2011)	France	51 carers	Spouse: 57.0	3.5	Alzheimer: 100.0	PIXEL Study	**Authoritarianism; Benevolence; Social restrictiveness; Community mental health ideology; Emotional Reaction Rejection; Emotional Reaction Anxiety; Emotional Reaction Agressiveness;** Emotional Reaction Prosocial Reactions; Perceived overall incompetence; Perceived susceptibility of having AD during one day
		Female %: 66.67	Daughter/son: 37.0		Severity: NR		
		Mean age (SD)	Sibilings: 2.0				
		Female: 64.3 (10.2)	Other: 10.0				
		Male: 74.5 (14.7)					

Abbreviations*:* AD, Alzheimer’s Disease; ADL, activities of daily living; BDI, Bender Depression Inventory; BPSD, Behavioral and psychological symptoms of dementia; CES-D, Center for Epidemiologic Studies Depression Scale; DBD, Dementia Behavior Disturbance; FBIS, Family Burden Interview Schedule; HRQoL VAS, Visual Analogue Rating Scale of Health-Related Quality of Life; IADL, Instrumental activities of daily living; MBPC, Memory and Behavior Problems Checklist; MBPC-R, Memory and Behaviour Problems Checklist-revised; NPI, Neuropsychiatric Inventory; NR, not reported; PwD, people with dementia; PHQ-9, Patient Health Questionnaire 9; QoL, quality of life; QoL-AD, quality of life in Alzheimer’s disease; SD, standard deviation; SF, Short form; WHO, World Health Organization; ZBI, Zarit Burden Interview; ZDRS, Zung Depression Rating Scale.

^a^ Variables in bold are those ones that presented statistically significant correlations with carer QoL.

#### Quality of Life measures

The most commonly used measure of carer QoL were Quality of Life in Alzheimer’s disease for carers^40^ (QoL-AD; n = 7), 36-Item Short Form Survey^41^ (SF-36; n = 6), and WHO-QOL-BREF^13^ (n = 6). Over 60% of the included studies (n = 20) used a general QoL measure (eg, QoL-AD and WHO-QOL-BREF), and the rest used a HRQoL measure (eg, SF-36, EuroQol-5D^42^).

#### Independent variables

Most of the included studies reported correlations between carer QoL and carer subjective burden (n = 11), carer depression (n = 10), people with dementia’s neuropsychiatric symptoms (n = 11), and their level of independence in activities of daily living (ADL; n = 10). The majority of the studies used the Zarit Burden Interview^43^ to measure subjective burden (n = 10), the Beck Depression Inventory^44^ to measure depression (n = 5), the Neuropsychiatric Inventory (NPI)^45^ to measure neuropsychiatric symptoms (n = 6), and the Katz Index of Activities of Daily Living^46^ (n = 3) to measure ADL.

Independent variables that were not included in the meta-analysis due to the number of studies identified were carer anxiety, satisfaction with life, coping strategies, social skills, frequency of nocturnal disruptions, relationship quality with the person with dementia, interpersonal support, some personality traits such as extraversion and neuroticism, physical health, number of hours providing care weekly, and duration of caregiving in years (see Table 1).

### Risk of Bias Within Studies

The assessment of study quality and bias using the Appraisal of Cross-sectional Studies tool is presented in Table 2. All of the included studies clearly specified the aim of the study, used the appropriate study design, clearly defined the target population, measured carer QoL appropriately, used validated questionnaires, fully described the methods, and presented the results of all the analyses described in the methods. Overall, the methodological quality was adequate across the included studies. However, the majority of the included studies (n = 25) did not justify the sample size, and almost no studies reported information about nonresponders.

**Table 2.** Assessment of Study Quality Using the Appraisal of Cross-Sectional Studies Tool.

	Study number according to Table 1																																
	1	2	3	4	5	6	7	8	9	10	11	12	13	14	15	16	17	18	19	20	21	22	23	24	25	26	27	28	29	30	31	32	33
Introduction																																	
Were the aims/objectives of the study clear?	Y	Y	Y	Y	Y	Y	Y	Y	Y	Y	Y	Y	Y	Y	Y	Y	Y	Y	Y	Y	Y	Y	Y	Y	Y	Y	Y	Y	Y	Y	Y	Y	Y
Was the study design appropriate for the stated aim(s)?	Y	Y	Y	Y	Y	Y	Y	Y	Y	Y	Y	Y	Y	Y	Y	Y	Y	Y	Y	Y	Y	Y	Y	Y	Y	Y	Y	Y	Y	Y	Y	Y	Y
Was the sample size justified?	Y	N	N	N	N	N	Y	N	N	Y	Y	Y	N	N	N	Y	N	N	N	N	Y	N	N	N	N	N	N	Y	N	N	N	N	N
Was the target/reference population clearly defined? (Is it clear who the research was about?)	Y	Y	Y	Y	Y	Y	Y	Y	Y	Y	Y	Y	Y	Y	Y	Y	Y	Y	Y	Y	Y	Y	Y	Y	Y	Y	Y	Y	Y	Y	Y	Y	Y
Was the sample frame taken from an appropriate population base so that it closely represented the target/reference population under investigation?	Y	Y	N	Y	Y	N	Y	Y	Y	Y	Y	Y	Y	N	N	Y	Y	Y	Y	Y	Y	Y	Y	Y	Y	N	N	Y	Y	Y	Y	Y	N
Was the selection process likely to select subjects/participants that were representative of the target/reference population under investigation?	Y	Y	N	N	N	Y	Y	Y	Y	Y	Y	Y	Y	Y	Y	Y	N	N	N	Y	N	N	N	Y	Y	N	Y	N	Y	Y	N	Y	Y
Were measures undertaken to address and categorize nonresponders?	Y	N	N	Y	Y	N	Y	N	Y	Y	N	N	N	Y	Y	Y	N	N	N	Y	Y	N	Y	Y	N	N	Y	N	N	N	N	N	N
Were the risk factor and outcome variables measured appropriate to the aims of the study?	Y	Y	Y	Y	Y	Y	Y	Y	Y	Y	Y	Y	Y	Y	Y	Y	Y	Y	Y	Y	Y	Y	Y	Y	Y	Y	Y	Y	Y	Y	Y	Y	Y
Were the risk factor and outcome variables measured correctly using instruments/measurements that had been trialled, piloted, or published previously?	Y	Y	Y	Y	Y	Y	Y	Y	Y	Y	Y	Y	Y	Y	Y	Y	Y	Y	Y	Y	Y	Y	Y	Y	Y	Y	Y	Y	Y	Y	Y	Y	Y
Is it clear what was used to determined statistical significance and/or precision estimates? (eg, *P* values, confidence intervals)	Y	Y	Y	Y	Y	Y	Y	Y	Y	Y	Y	Y	Y	Y	Y	Y	Y	Y	Y	Y	Y	Y	Y	Y	Y	Y	Y	Y	Y	Y	Y	Y	Y
Were the methods (including statistical methods) sufficiently described to enable them to be repeated?	Y	Y	Y	Y	Y	Y	Y	Y	Y	Y	Y	Y	Y	Y	Y	Y	Y	Y	Y	Y	Y	Y	Y	Y	Y	Y	Y	Y	Y	Y	Y	Y	Y
Results																																	
Were the basic data adequately described?	Y	Y	Y	Y	Y	Y	Y	Y	Y	Y	Y	N	Y	N	Y	Y	Y	Y	Y	Y	Y	Y	Y	Y	Y	Y	Y	Y	Y	Y	N	Y	Y
Does the response rate raise concerns about nonresponse bias?	Y	N	N	Y	Y	N	Y	N	Y	N	N	N	N	Y	Y	Y	N	N	N	Y	N	N	Y	Y	N	N	Y	N	N	N	N	N	N
If appropriate, was information about nonresponders described?	N	N	N	N	N	N	Y	N	N	N	N	N	N	N	N	N	N	N	N	N	N	N	N	N	N	N	N	N	N	N	N	N	N
Were the results internally consistent?	Y	Y	Y	Y	Y	Y	Y	Y	Y	Y	Y	N	Y	Y	Y	Y	Y	Y	Y	Y	Y	Y	Y	Y	Y	Y	Y	Y	Y	Y	Y	Y	Y
Were the results presented for all the analyses described in the methods?	Y	Y	Y	Y	Y	Y	Y	Y	Y	Y	Y	Y	Y	Y	Y	Y	Y	Y	Y	Y	Y	Y	Y	Y	Y	Y	Y	Y	Y	Y	Y	Y	Y
Discussions																																	
Were the authors’ discussions and conclusions justified by the results?	Y	Y	Y	Y	Y	Y	Y	Y	Y	Y	Y	Y	Y	Y	Y	Y	Y	Y	Y	Y	Y	Y	N	Y	Y	Y	Y	Y	Y	Y	Y	Y	Y
Were the limitations of the study discussed?	Y	N	Y	Y	Y	Y	Y	N	Y	Y	Y	Y	Y	Y	Y	Y	Y	Y	Y	Y	Y	Y	N	Y	Y	N	Y	Y	N	N	N	Y	Y
Other																																	
Were there any funding sources or conflicts of interest that may affect the authors’ interpretation of the results?	Y	N	N	Y	N	Y	Y	Y	Y	Y	N	Y	N	N	N	N	N	Y	Y	Y	Y	N	Y	N	Y	N	Y	Y	N	Y	Y	N	Y
Was ethical approval or consent of participants attained?	Y	Y	Y	N	Y	Y	Y	Y	Y	Y	N	Y	N	N	Y	Y	Y	Y	Y	Y	Y	Y	Y	Y	Y	Y	Y	Y	Y	Y	Y	Y	N
Total number of items rated as yes	19	14	13	16	16	15	20	15	18	18	15	15	14	14	16	18	14	15	15	18	17	14	15	17	16	12	17	16	14	15	13	15	14

Abbreviations*:* Y, Yes (the study clearly demonstrated the information regarding the question); N, No (no clear information was provided in the study to record the item as yes).

### Synthesis of Results

Twenty-seven studies included in the meta-analysis demonstrated associations between carer QoL and different types of carer-related independent variables (subjective burden, depression, age, income, and distress) and people with dementia-related independent variables (neuropsychiatric symptoms, ADL, cognitive functioning, and self-/proxy-rated QoL). A random model was used for carer depression and subjective burden, people with dementia’s proxy-rated QoL, their neuropsychiatric symptoms, and ADL due to significant heterogeneity.

### Independent Variables With a Significant Effect Size

#### Carer’s depression (number of studies included in the analysis n = 10)

**Figure 2.**
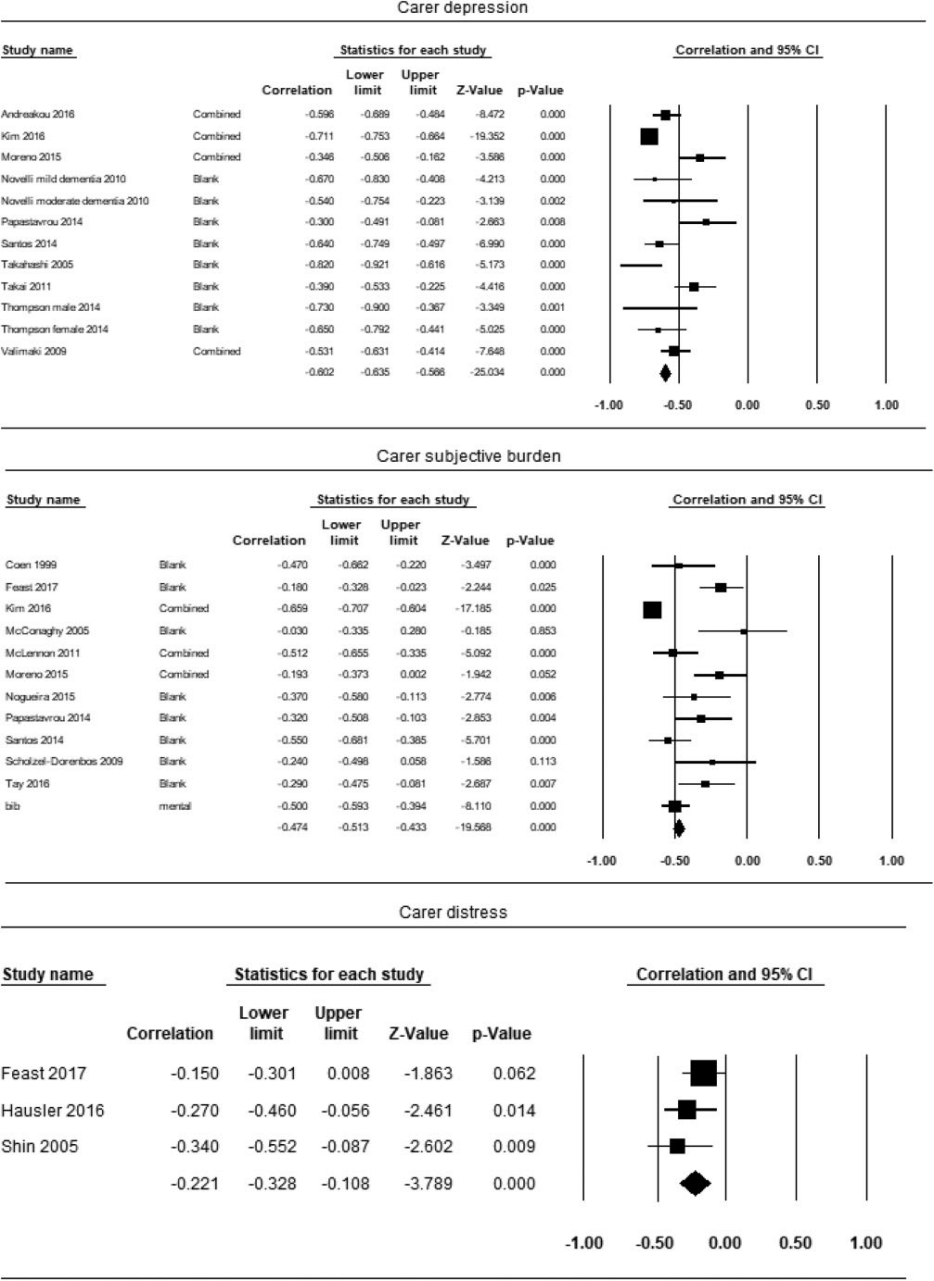

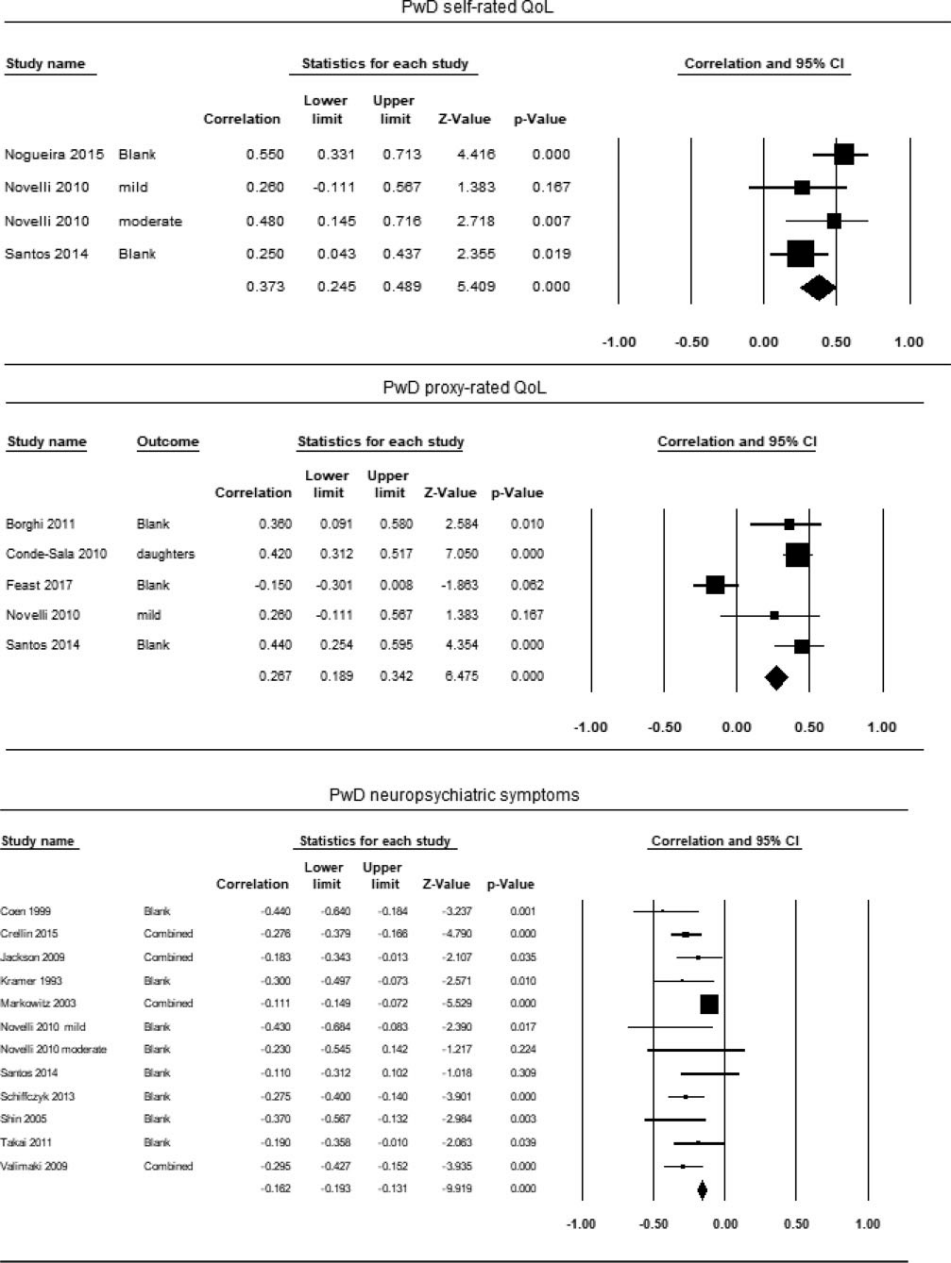
Forest plot for independent variables with a significant effect.

Ten studies reported the correlation coefficient between carer QoL and depression (Figure 2). The effect sizes varied from −0.30 to −0.82. Overall, the point estimate of effect size between carer QoL and depression was −0.58 (95% CI = −0.66 to −0.48, *P* < .00), suggesting a significant large effect. There was statistically significant high heterogeneity between study effect sizes (*I*^2^ = 80.77%, *Q* = 57.29).

#### Carer’s subjective burden (n = 11)

The effect sizes varied from −0.03 to −0.66. The point estimate of effect size between carer QoL and subjective burden was −0.47 (95% CI = −0.51 to −0.21, *P* < .00), suggesting a significant moderate effect. The heterogeneity between study effect sizes was significantly high (*I*^2^ = 87.95%, *Q* = 82.98).

#### Carer’s distress (n = 3)

The effect sizes varied from −0.15 to −0.34. The point estimate of effect size between carer QoL and care’s distress was small −0.22 (95% CI = −0.33 to −0.11, *P* < 0.00). The heterogeneity between study effect sizes was not significant (*I*^2^ = 0.00%, *Q* = 1.94). However, this could be due to the limited number of studies included.

#### People with dementia’s self-rated QoL (n = 3)

The effect sizes varied from 0.25 to 0.55. The point estimate of effect size between carer QoL and self-rated QoL was 0.37 (95% CI = 0.24 to 0.49, *P* < 0.00), suggesting a significant moderate effect. The heterogeneity between study effect sizes was not statistically significant (*I*^2^ = 41.07%, *Q* = 5.09).

#### People with dementia proxy-rated QoL (n = 5)

The effect sizes varied from −0.15 to 0.44. The point estimate of effect size between carer QoL and proxy-rated QoL was 0.27 (95% CI = −0.00 to 0.51, *P* < .05), suggesting a significant small effect. The heterogeneity between study effect sizes was significantly high (*I*^2^ = 89.69%, *Q* =38.79).

#### People with dementia’s neuropsychiatric symptoms (n = 11)

The effect sizes varied from −0.11 to −0.44. The point estimate of effect size between carer QoL and neuropsychiatric symptoms was −0.24 (95% CI = −0.31 to −0.17, *P* < .00), suggesting a significant small effect. There was statistically significant moderate heterogeneity between study effect sizes (*I*^2^ = 61.77%, *Q* = 28.73).

### Independent Variables With No Significant Effect Size

#### Carer’s income (n = 4)

The effect sizes varied from −0.06 to 0.30 (Supplementary Figure 1). The point estimate of effect size between carer QoL and care’s income was 0.13 (95% CI = −0.00 to 0.26, *P* = .06). Both the overall effect size and the heterogeneity between study effect sizes were not statistically significant (*I*^2^ = 42.23%, *Q* = 5.19).

#### Carer’s age (n = 10)

The effect sizes varied from −0.10 to 0.10. Overall, the point estimate of effect size between carer QoL and carer’s age was −0.03 (95% CI = −0.05 to 0.0, *P* = .13). Both the overall effect size and the heterogeneity between study effect sizes were not statistically significant (*I*^2^ = 0.00%, *Q* = 2.58).

#### People with dementia cognitive functioning (n = 8)

The effect sizes varied from −0.15 to 0.29. The point estimate of effect size between carer QoL and cognitive functioning was −0.04 (95% CI = −0.05 to 0.13, *P* = 0.40). Both the overall effect size and the heterogeneity between study effect sizes were not statistically significant (*I*^2^ = 44.83%, *Q* = 14.50).

#### People with dementia ADL (n = 10)

The effect sizes varied from−0.33 to 0.17. The point estimate of effect size between carer QoL and ADL was −0.01 (95% CI = −0.07 to 0.8, *P* = .79). Both the overall effect size and the heterogeneity between study effect sizes were not statistically significant (*I*^2^ = 53.20%, *Q* = 21.37).

### Risk of Bias Across Studies

The Duval and Tweedie trim-and-fill approach suggested that potentially no studies are missing for carer’s depression, distress, income, and age as well as people with dementia’s neuropsychiatric symptoms and ADL. The results demonstrated that 6 studies are potentially missing for carer’s subjective burden and 3 for people with dementia’s cognitive functioning. If these missing studies were imputed, the point of estimate would decrease to −0.58 (95% CI = −0.69 to −0.44) and −0.01 (95% CI = −0.07 to 0.05), respectively. The results demonstrated that one study is potentially missing for people with dementia’s self-rated and proxy-rated QoL. If these studies are imputed, the point of estimate would decrease to 0.30 (95% CI = 0.18 to 0.41) and 0.23 (95% CI = −0.01 to 0.44), respectively.

Rosenthal’s Fail-safe *N* analysis suggested that more than 100 studies are required for the combined 2-tailed *P* value to exceed .05 for depression, subjective burden, and people with dementia’s neuropsychiatric symptoms, suggesting that the observed point of estimates are likely to be robust for these independent variables. Rosenthal’s Fail-safe *N* analysis suggested that less than 50 studies are required for carer’s distress people with dementia’s self-rated QoL and proxy-rated QoL suggesting that the observed point of estimates are less likely to be robust for these 2 variables.

### Subgroup Analyses

Subgroup analyses were conducted with independent variables, which demonstrated a significant heterogeneity (ie, people with dementia’s neuropsychiatric symptoms, their proxy-rated QoL, carer’s depression, and carer’s subjective burden). The possible sources of variance were tested using 3 moderators (ie, the development status of the country, types of measures used to assess carer QoL, and types of measures used to assess the independent variable).

#### People with dementia neuropsychiatric symptoms

Subgroup analyses demonstrated that the point of estimate for neuropsychiatric symptoms differed according to the type of measure used to assess neuropsychiatric symptoms (*P* < .01) but not according to the development status of the country (*P* = .79) or the type of measures used to assess carer QoL (*P* = .47). The subgroup of studies that used Revised Memory and Behaviour Problems Checklist^47^ reported the lowest effect estimate, while the study that used the Baumgarten Dementia Behaviour Disturbance questionnaire (DBD)^48^ reported the highest estimate of effect.

#### People with dementia’s proxy-rated QoL

Subgroup analyses demonstrated that the point of estimate for people with dementia’s proxy-rated QoL differed according to the type of measure used to assess their QoL (*P* <.01) and the types of measures used to assess carer QoL (*P* < .01) but not according to the development status of the country (*P* = .48). The subgroup of studies that used EQ-5D to assess proxy-rated QoL as an independent variable reported the lowest effect estimate, while the studies that used proxy-rated QoL-AD reported the highest estimate of effect. The subgroup of studies that used EQ-5D to assess carer QoL as a dependent variable reported the lowest effect estimate, while the studies that used SF-12 reported the highest estimate of effect.

#### Carer’s depression

The test for subgroup differences indicated that the point of estimate for carer’s depression did not differ according to any of moderators (measures used to assess depression *P* = .72; measures used to assess carer QoL *P* = .94; development status of the country *P* = .69).

#### Carer’s subjective burden

Subgroup analyses demonstrated that the point of estimate for carer’s subjective burden did not differ according to any of moderators (measures used to assess subjective burden *P* = .68; measures used to assess carer QoL *P* = 4.00; development status of the country *P* = .48).

## Discussion

The current meta-analysis had 2 purposes, mainly to quantify the point estimate of effect size between carer QoL and different types of independent variables related to carers themselves and people with dementia. Secondly, it aimed to explore factors that may moderate the strength of such relationships, including the development status of the country and types of tools used to assess the measures of interest. To our knowledge, this was the first meta-analysis to quantitatively synthesize the factors associated with carer QoL. Thirty-three cross-sectional studies providing data from 6177 family carers were included; however, only 27 studies were included in the final meta-analysis.

The current meta-analysis found that the pooled correlations with carer QoL (ie, effect size) were significantly large for depression and significantly moderate for carer subjective burden, while the effect size for people with dementia’s neuropsychiatric symptoms was significant but small. These results were indicated to be robust in the context of publication bias. The effect size for people with dementia’s self-rated QoL was also significantly moderate. Furthermore, the effect size was significantly small for people with dementia’s proxy-rated QoL and carer’s distress. However, these results were less likely to be robust in the context of publication bias; therefore, the findings need to be interpreted with caution.

The results of this meta-analysis support evidence from the previous review,^21^ which suggested that carer’s mental health and people with dementia’s behavioral and psychological symptoms were strongly associated with carer QoL. On the other hand, the findings differed from those of de Oliveira et al, which included only studies that targeted carers aged 60 and over.^22^ While the previous review suggested that carer’s increased age was associated with lower levels of QoL, the results of the current meta-analysis without any age restriction did not support this association. This could be due to the differences in methodological approaches. De Oliveira et al included both regression and correlational studies in the systematic review and did not conduct a quantitative synthesis.^22^ The current study also included 4 studies that were not considered in the review conducted by de Oliveira et al, and the findings of the current study were similar to those from a more recent review conducted by Farina et al, which concluded that the associations between carer QoL and carer age to be less clear.^21^

The results of subgroup analyses demonstrated the moderating effect of the country development status (ie, high vs very high developed countries) was not significant for any of the independent variables. The results of subgroup analyses suggest that independent variables, which are considered to be a critical predictor of carer QoL (ie, carer depression, carer subjective burden, and neuropsychiatric symptoms) may be important variables for intervention regardless of the opportunities offered for better health, education, and living conditions across different high and very high developed countries.

This finding is particularly important as, in the recent years, there has been an increase in the number of interventions developed for family carers of people with dementia, but the majority of well-established interventions have only been tested in the most economically developed countries.^49,50^ Interventions that can be accessed globally and can support carers worldwide are urgently needed considering that a greater number of people with dementia are currently living in low- and middle-income countries, and this trend is expected to be more profound in the future.^51^

The well-established multicomponent interventions that can tackle some of the critical predictors such as START^52^ could be beneficial for carers from countries with the lower development status if the intervention materials could be translated into multiple languages. However, there are other factors that should be considered apart from the language translation such as differences in culture, health, and social care systems and the availability of resources including skilled therapists. To address such challenges, the 10/66 Dementia Research Group developed a program called Helping Carers to Care, which is a psychoeducational intervention especially designed for use in low- and middle-income countries, and this program has already been tested in India, Peru, and Russia.^53^

The results of subgroup analyses also demonstrated that the type of measure used to assess independent variables such as neuropsychiatric symptoms, and people with dementia’s proxy-rated QoL may moderate the relationship between these variables and carer QoL. It is not possible to make direct recommendations on which measures to be used to assess these types of variables based on the current review due to a large variability across included studies. The future studies are required to carefully make a choice of measures guided by several considerations, such as the setting in which the assessment will occur and their reliability and validity. For example, previous studies have found that the NPI seems to be one of the most efficient measures of people with dementia’s neuropsychiatric symptoms, as it includes multiple behavioral domains at a general level as well as targets-specific behaviors within domains and can be used in multiple clinical settings.^54^ A recent systematic review, which identified 16 different types of QoL measures specifically designed for people with dementia, concluded that many measures still have limited evidence supporting their reliability and validity, and thus more research is needed to have complete confidence in their utility.^55^

### Limitations

This meta-analysis has some methodological limitations. Firstly, although we made every effort to minimize missing studies, all the identified studies were from high or very high developed countries as indicated by the HDI category. Regardless of the inclusion of non-English articles, the current meta-analysis was not able to identify any studies from low developed countries (eg, countries from Africa, Central America, Caribbean islands, and some areas of Asia). However, it is worth mentioning that the current meta-analysis included 7 studies conducted in countries that are defined as high developed countries by the HDI (eg, Colombia and Brazil) but are also considered middle-income countries according to the World Bank classification by income per capita.^56^ Thus, the results of the subgroup analysis by the HDI category still provide an important implication. It is recommended future cross-sectional studies focus on researching the impact of caring on carer QoL in low developed countries, as a great number of people with dementia are expected to be living in these countries.^57^

Secondly, due to a large variation in the existing assessment tools, it was not possible to have enough studies in each subcategory when conducting subgroup analyses for some independent variables such as people with dementia’s proxy-rated QoL and their neuropsychiatric symptoms. For example, 11 studies with 4 different types of measures were included in the analysis of neuropsychiatric symptoms. Of these 11 studies, there was only one study that used the DBD. Consequently, these results could potentially change if more studies are included.

Furthermore, subgroup analyses were also challenging, as characteristics of the sample (eg, relationship with the person with dementia, and hours of caring per day) were not fully reported across the included studies. Therefore, only 3 moderating factors were explored in the current study. In order to conduct a robust moderation analysis, we encourage future cross-sectional studies to fully report data on sample characteristics for both carers and people with dementia.

Thirdly, similar to previous reviews,^21-23^ all included studies employed a generic QoL or HRQoL measures to assess carer QoL, and no studies used care-related QoL measures. This is problematic, as generic measures of QoL may not capture caring-specific components that can affect QoL and might not be sensitive enough for detecting changes in the progression of dementia.^21,58,59^ Therefore, it is recommended that future studies use carer-related QoL instruments.

Fourthly, some independent variables that reported a statistically significant correlation with carer QoL were not included in the meta-analysis due to the small number of studies identified (ie, fewer than 3 studies). These independent variables included carer anxiety, satisfaction with life, coping strategies, social skills, frequency of nocturnal disruptions, relationship quality with the person with dementia, interpersonal support, some personality traits such as extraversion and neuroticism, physical health, number of hours providing care weekly, and duration of caregiving in years. Future studies should continue exploring the association of carer QoL with these variables in order to be included in future meta-analyses, especially with anxiety as the correlation was reported to be strong in two studies.^60,61^ A recent systematic review also highlighted that although anxiety is a prevalent psychological difficulty experienced by family carers of people with dementia, it is somewhat neglected compared to other carer outcomes (eg, care burden, depression) in the current literature and therefore requires more attention.^62^

Previous studies also have demonstrated that carer’s race and ethnicity can have an impact on carer outcomes such as depression and burden.^63-65^ Ethnicity was not included in the current meta-analysis, as in most of the included studies the data were collected mainly from white carers, and there was a lack of diversity in the study samples. Future cross-sectional studies should look at other ethnicities and races to understand how it might affect the caring experience.

Finally, the current meta-analysis was based on correlational studies, and thus, the causality in the relationship between independent and dependent variables may not be entirely one way. It is possible that poorer carer QoL could lead to higher depression or worse neuropsychiatric symptoms. Future longitudinal studies should explore how these variables change over time as dementia progresses.

## Conclusion and Implications

In summary, this meta-analysis revealed that carer depression, carer subjective burden, and people with dementia’s neuropsychiatric symptoms are critical predictors of carer QoL. Therefore, carer interventions that can target multiple outcomes, such as these 3 variables, seem important for improving carer QoL. Most of the included participants were female, over 55 years old, and from developed countries; thus, the findings may not be able to generalize to the groups of carers who do not fall into this category.

It is highly recommended for future studies to target a wider population, including those from low or moderately developed countries, to use instruments specifically designed for carers to measure carer QoL and to explore the relationship between carer QoL and those independent variables that seem to have a strong correlation with carer QoL but have been less studied such as carer anxiety.

## Supplemental Material

Supplementary_tables_and_figures - Factors Related to the Quality of Life in Family Carers of People With Dementia: A Meta-Analysis Click here for additional data file. Supplementary_tables_and_figures for Factors Related to the Quality of Life in Family Carers of People With Dementia: A Meta-Analysis by Milena L. Contreras, Eneida Mioshi and Naoko Kishita in Journal of Geriatric Psychiatry and Neurology
